# RETRACTION: Classic Prescription, Kai-Xin-San, Ameliorates Alzheimer's Disease as an Effective Multitarget Treatment: From Neurotransmitter to Protein Signaling Pathway

**DOI:** 10.1155/omcl/9868970

**Published:** 2025-06-10

**Authors:** Oxidative Medicine and Cellular Longevity

RETRACTION: S. Guo, J. Wang, H. Xu, et al., “Classic Prescription, Kai-Xin-San, Ameliorates Alzheimer's Disease as an Effective Multitarget Treatment: From Neurotransmitter to Protein Signaling Pathway,” *Oxidative Medicine and Cellular Longevity* 2019, no. 1 (2019): 1–14, https://doi.org/10.1155/2019/9096409.

The above article, published online on 01 July 2019 in Wiley Online Library (wileyonlinelibrary.com), has been retracted following the agreement of the journal's Chief Editor Jeanette Vazquez-Vivar; and John Wiley & Sons Ltd.

The retraction has been agreed following an investigation of the concerns raised by *Mycosphaerella arachidis* and *Indigofera tanganyikensis* on PubPeer [1], which identified concerns regarding Figure 4.

Specifically, similarity has been identified between the KXS panel shown in Figure 4d and the panel corresponding to PR – treated cells in Figure 4a of [2].

As a result of the investigation, the data and conclusions of this article are considered unreliable.

The authors disagree with the wording of this retraction notice.
